# Comparison of newly diagnosed COPD patients and the non-COPD residents in Shanghai Minhang District

**DOI:** 10.3389/fpubh.2023.1102509

**Published:** 2023-03-01

**Authors:** Xin Yin, Zixuan Zheng, Yue Dong, Junqing Li, Shuang Yang, Qian Xu, Shanshan Hou, Yi Zang, Heyuan Ding, Juan Xie, Zhijun Jie, Qingwu Jiang, Jindong Shi, Na Wang

**Affiliations:** ^1^Department of Epidemiology, School of Public Health, Fudan University, Shanghai, China; ^2^Department of Respiratory and Critical Care Medicine, Shanghai Fifth People‘s Hospital, Fudan University, Shanghai, China; ^3^Center of Community-Based Health Research, Fudan University, Shanghai, China; ^4^Lingang Laboratory, Shanghai, China; ^5^Department of General Medicine, Jiangchuan Community Healthcare Service Center of Minhang District, Shanghai, China; ^6^Center for Disease Control and Prevention of Xuhui District, Shanghai, China; ^7^Department of Endocrinology, Shanghai Fifth People's Hospital, Fudan University, Shanghai, China; ^8^Department of General Medicine, Shanghai Fifth People's Hospital, Fudan University, Shanghai, China

**Keywords:** chronic obstructive pulmonary disease, spirometry, screening, asymptomatic, general population

## Abstract

**Background:**

To compare whether the general population, especially those without characteristic symptoms, need spirometry screening for chronic obstructive pulmonary disease (COPD).

**Methods:**

Residents aged > 40 years old in Minhang, Shanghai, China, filled out screening questionnaires and underwent spirometry. The structured questionnaire integrating COPD population screening and COPD screening questionnaire was designed to obtain data on demographic characteristics, risk factors of COPD, respiratory symptoms, lifestyle habits, and comorbidities. We assessed the correlations between variables and COPD and the impact factors of FEV_1_% predicted.

**Results:**

A total of 1,147 residents were included with a newly diagnosed mild to moderate COPD prevalence of 9.4% (108/1,147); half of the patients (54/108) were asymptomatic. Multivariate analysis did not reveal any significant differences in symptoms or lifestyle factors between newly diagnosed COPD patients and non-COPD participants. However, according to the generalized linear model, older age (β = −0.062, *p* < 0.001), male sex (β = −0.031, *p* = 0.047), and respiratory symptoms (β = −0.025, *p* = 0.013) were associated with more severe airflow limitation.

**Conclusion:**

Newly diagnosed COPD patients had few differences compared with the general population, which suggests that a targeted case finding strategy other than general screening was currently preferred. More attention should be paid to respiratory symptoms when making a diagnosis and exploring new therapies and interventions for COPD in the early stage.

## 1. Introduction

Chronic Obstructive Pulmonary Disease (COPD) is a common chronic respiratory disease (1), which represents a serious public health challenge due to its increasing prevalence and related disability and mortality affecting individuals worldwide (2). The China Pulmonary Health (CPH) study, a national survey conducted in 2018 and comprising adults aged > 20 years from 10 provinces, revealed that the overall prevalence in adults aged > 20 and >40 years old was 8.6 and 13.7%, respectively (2). The Global Burden of Diseases Study (GBD), which was conducted in 2017 and included 195 countries, reported 3.2 million deaths due to COPD, classifying COPD as the seventh leading cause of years of life lost (YLLs) (3, 4).

Early detection, diagnosis, and intervention should be the main approaches to the prevention of COPD (5). Global Initiative for Chronic Obstructive Lung Disease (GOLD) Committee recommends that anyone with characteristic symptoms, such as dyspnea, chronic cough, and sputum production or a history of long-term exposure to risk factors, with especial emphasis on individuals aged > 40 years old, should undergo COPD screening (1). GOLD 2022 indicates that a forced expiratory volume in the first second to forced vital capacity (FEV1/FVC) ratio of <0.70 based on post-bronchodilator spirometry confirmed the presence of persistent airflow limitation. In fact, this is the current diagnostic criterion (1). However, screening spirometry in the general population is somewhat controversial. In 2022, the US Preventive Services Task Force (USPSTF) argued that there is still no evidence that screening for COPD in asymptomatic patients positively affects the health-related quality of life (6, 7). Given the lack of experienced spirometry examiners, the confusion related to diagnosis criterion, and the low utilization of bronchodilators before the screening, many spirometry procedures failed to meet the guidelines, resulting in serious underdiagnoses and misdiagnoses of COPD (8, 9). Moreover, false-positive reports due to improper operation might have adverse consequences, such as unreasonable anxiety and/or depression (10).

In order to compare whether the general population, especially those without characteristic symptoms, need spirometry screening, we conducted a screening of residents aged > 40 years old in Minhang District, Shanghai (China) by using a questionnaire and spirometry, which were then analyzed to assess the differences in demographic characteristics, symptoms, lifestyles, and disease comorbidities between non-COPD residents and newly diagnosed mild to moderate COPD patients.

## 2. Materials and methods

### 2.1. Participants

From November 2019 to August 2020, participants in Minhang District, Shanghai, China, were selected based on the following inclusion criteria: (1) resided in Jiangchuan, Maqiao, or Wujing Town for more than 10 years; (2) aged ≥ 40 years old; (3) signed informed consent. Individuals were excluded if they: (1) had relative contraindications in Standardization of Spirometry 2019 Update (11); (2) could not understand the questionnaire or cooperate with spirometry. Besides, previously diagnosed COPD patients or those with COPD in GOLD stage 3 or 4 based on the current screening were excluded from the analysis.

The study was evaluated and approved by the Ethics Committee of Shanghai Fifth people's Hospital (2018 Ethics Approval No. 130). All participants signed informed consent, and all personal information was de-identified before further analysis.

### 2.2. Questionnaire investigation

A structured questionnaire was designed by integrating the commonly used screening scales, such as COPD population screening (COPD-PS) and COPD screening questionnaire (COPD-SQ) (12–14). The following information was collected: (1)demographic characteristics, including sex, age, education, etc.; (2) risk factors of COPD, including smoking, body mass index (BMI), family history of respiratory disease, etc.; (3) respiratory symptoms including chronic cough (chough lasting 3 months for 2 consecutive years), dyspnea, phlegm, limited activities, and the modified Medical Research Council (mMRC) scale were included for assessment of symptoms; (4) lifestyle habits including smoking, biomass smoke exposure, and sleep quality [sleep quality was categorized by Pittsburgh sleep quality index (PSQI) scores and insomnia defined as a global score of >5 (15)]; (5) comorbidities including asthma, tuberculosis, diabetes, and similar.

Interviewers were respiratory physicians from Shanghai Fifth People's Hospital or general practitioners of Minhang Community Health Service Center, who received unified training and passed an assessment before the investigation.

### 2.3. Spirometry test

Trained operators conducted spirometry by using MicroQuark-PONY FX spirometers, Spirometry was performed following the procedures recommended by Standardization of Spirometry 2019 Update (11). Environment and volume calibration were undertaken every day, and linear calibration was undertaken once a week. Each participant's age, height, and weight were recorded before spirometry. If FEV_1_/FVC < 0.7, 400 ug Salbutamol Sulfate Aerosol (Ventolin) was used to dilate bronchus in the short term, and spirometry was done after 15 min. According to 2022 GOLD, the fixed FEV_1_/FVC ratio was used to diagnose COPD, and the classification of airflow limitation severity was based on FEV_1_%predicted (FEV_1_%pred): GOLD stage 1 (mild COPD) corresponding to FEV_1_ ≥ 80% pred, GOLD stage 2 (moderate COPD) corresponding to FEV_1_ 50–80% pred (1).

### 2.4. Statistical analysis

SPSS v.23.0 (IBM) was used for statistical analysis, and figures were drawn by OriginPro v.2022. Continuous variables were categorized and reported as frequency (*n*) or rate (%) along with other categorical variables. Categorical variables were compared between COPD and non-COPD participants by using the Chi-square test. Other categorical variables were compared by Fisher's exact test. Sleep latency and PSQI scores were reported as medians and compared by a non-parametric test due to non-normal distribution. Multivariate logistic regression analysis was performed to evaluate correlations between COPD and other variables. Odds ratios (OR) and 95% confidence intervals (95%CI) were estimated. The variables that were common risk factors for COPD and whose *p*-value < 0.2 in univariate analysis were entered. The generalized linear mode was performed to assess impact factors of FEV_1_%pred. A two-tailed *p*-value of <0.05 was considered statistically significant.

## 3. Results

### 3.1. Demographic characteristics

**Figure 1:**
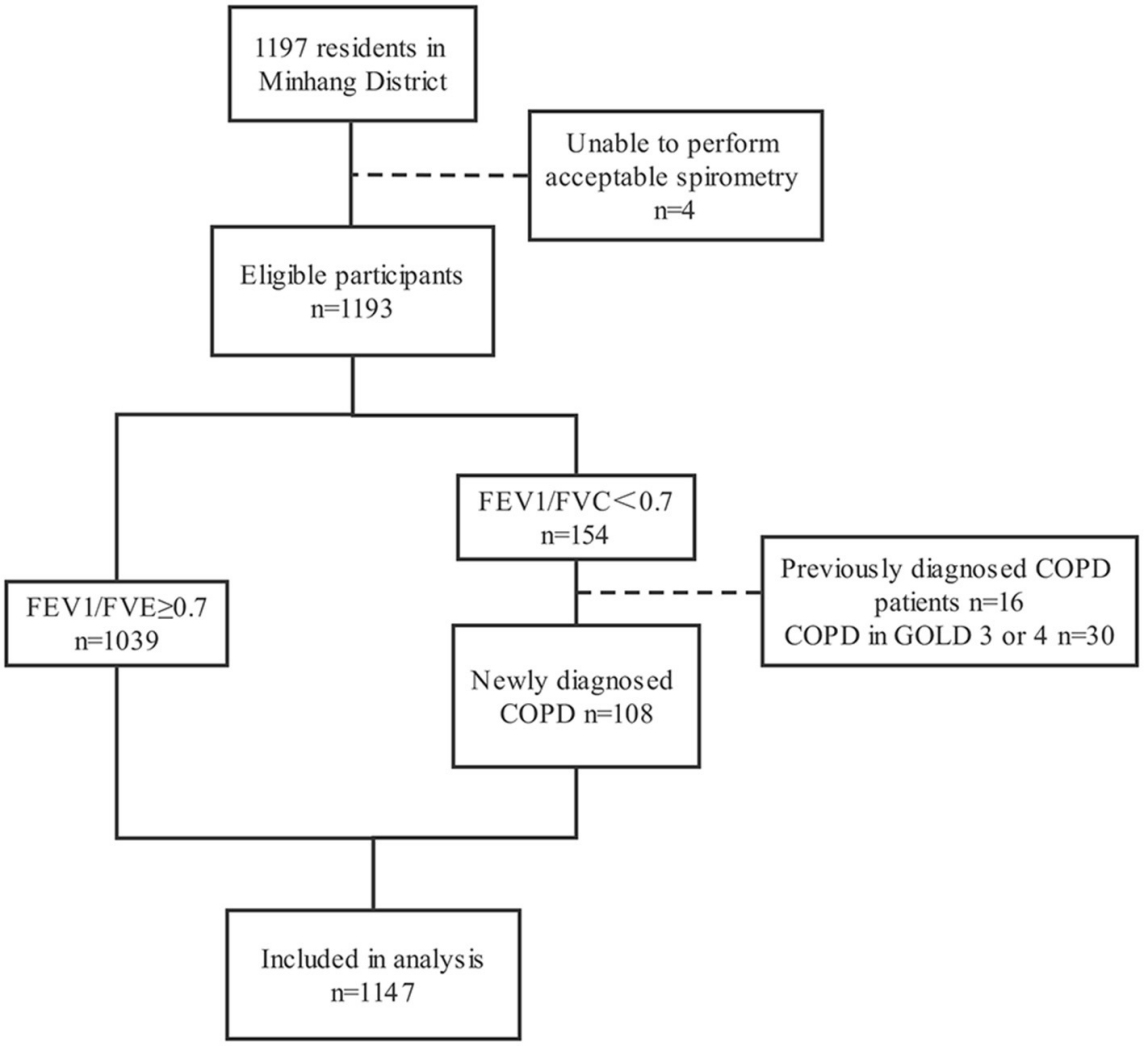
Study flow chart.

A total of 1,197 participants completed the questionnaire and spirometry test. The prevalence of COPD in 1,197 participants was 12.9% (154/1,197), and the self-reported prevalence was 10.4% (16/154). Four participants with unqualified spirometry tests, 16 previously diagnosed COPD patients, and 30 patients in GOLD stage 3 or 4 were excluded, resulting in 1,147 participants in the current analysis. The detailed process is shown in Figure 1.

Among 1,147 participants, there were 479 (41.8%) males and 66 8 (58.2%) females. The mean age of participants was 67.5 (SD: 9.7), and those aged ≥ 60 accounted for 80.8%. One hundred eight (9.4%) participants were newly diagnosed as mild to moderate COPD patients based on the GOLD spirometric criteria, with the corresponding prevalence of 13.8% (66/479) in males and 6.3% (42/668) in females (*P* < 0.001). Among newly diagnosed patients, the proportion of mild COPD patients was 38.9% (42/108) and moderate COPD patients 61.1% (66/108). The demographic characteristics of all participants are summarized in Table 1. As compared with non-COPD participants, COPD patients were more likely to be males and older. However, there were no significant differences in BMI, education, and family history of respiratory diseases between the two groups (all *P* > 0.05) (Table 1).

**Table 1:** Demographic characteristics of 1,147 participants with/without COPD.

	**Total**	**Non-COPD**	**Mild to moderate COPD**	***P*** **–value**
*N* (%)	1,147 (100.0)	1,039 (90.6)	108 (9.4)	
Sex				<0.001
Male	479 (41.8)	413 (86.2)	66 (13.8)	
Female	668 (58.2)	626 (93.7)	42 (6.3)	
Age				<0.001
40–59	220 (19.2)	212 (96.4)	8 (3.6)	
60–69	459 (40.0)	419 (91.3)	40 (8.7)	
≥70	468 (40.8)	408 (87.2)	60 (12.8)	
BMI				0.567
< 18.5	50 (4.4)	45 (90.0)	5 (10.0)	
18.5–23.9	557 (48.6)	503 (90.3)	54 (9.7)	
24–27.9	446 (38.9)	402 (90.1)	44 (9.9)	
≥28	94 (8.2)	89 (94.7)	5 (5.3)	
Education				0.294
Never	125 (10.9)	116 (92.8)	9 (7.2)	
< 9 years	775 (67.6)	694 (89.7)	81 (10.3)	
≥9 years	247 (21.5)	229 (92.4)	18 (7.6)	
Family history of respiratory diseases				0.792
Yes	119 (10.4)	107 (89.9)	12 (11.2)	
No	1,028 (89.6)	932 (90.7)	96 (9.3)	

### 3.2. Respiratory symptoms of participants with/without COPD

As shown in Table 2, in mild to moderate COPD patients, only 7.4% (8/108) had a chronic cough lasting 3 months for 2 consecutive years; 10.2% (11/108) coughed up phlegm; 36.1% (39/108) suffered from shortness of breath; 18.5% (20/108) experienced activity limitation because of respiratory problems. According to mMRC, only 10.2% (11/108) of patients reported walking slower than other people of the same age or needed to stop to take a breath after walking 100 yards. Overall, there were 34.3% (37/108) of patients with one of the 4 kinds of symptoms mentioned above and 15.7% of patients (17/108) with two or more symptoms. Nevertheless, there were 54 asymptomatic patients, accounting for half of the total patients. Newly diagnosed COPD patients and non-COPD participants had no significant difference in common symptoms of COPD, including cough, expectoration, shortness of breath, and activity limitation (*P* > 0.05).

**Table 2:** Respiratory symptoms of 1,147 participants.

	**Total**	**Non-COPD**	**Mild to moderate COPD**	***P*** **-value**
Chronic cough				0.762
Yes	77 (6.7)	69 (6.6)	8 (7.4)	
No	1,070 (93.3)	970 (93.4)	100 (92.6)	
Dyspnea				0.502
Yes	381 (33.2)	342 (32.9)	39 (36.1)	
No	766 (66.8)	697 (67.1)	69 (63.9)	
Phlegm				0.217
Yes	162 (14.1)	151 (14.5)	11 (10.2)	
No	985 (85.9)	888 (85.5)	97 (89.8)	
Limited activity				0.537
Yes	243 (21.2)	223 (21.5)	20 (18.5)	
No	904 (78.8)	816 (78.5)	88 (81.5)	
mMRC scores				0.588
0	851 (74.2)	774 (74.5)	77 (71.3)	
1	171 (14.9)	151 (14.5)	20 (18.5)	
2	73 (6.4)	66 (6.4)	7 (6.5)	
3	37 (3.2)	33 (3.2)	4 (3.7)	
4	15 (1.3)	15 (1.54)	0	

### 3.3. Living behaviors of participants with/without COPD

A total of 165 (14.4%) participants smoked over 30 pack-years, and the prevalence of mild to moderate COPD in this population was 15.8%, which was higher than that in the total participants. The prevalence in current smokers (18.0%) was also higher than that in those who never smoked (6.9%) or in former smokers (10.5%). Three hundred eighty-four (33.5%) participants reported being exposed to dust in the workplace. The median time for sleep latency for all participants was 15 min. The median sleep efficiency for COPD patients was 91%, which was similar with non-patients. According to PSQI scores, 28.7% (31/108) patients had poor sleep quality. Univariate analysis showed that COPD patients had better sleep quality than other participants. There were no significant differences in occupational dust exposure, biofuel exposure, sleep latency, and sleep efficiency between the two groups (all *P* > 0.05) (Table 3).

**Table 3:** Living behaviors of 1,147 of participants with/without COPD.

	**Total**	**Non-COPD**	**Mild to moderate COPD**	***P*** **-value**
Smoking pack-years				<0.001
Never smoke	806 (70.3)	750 (93.1)	56 (6.9)	
<30 (pack-years)	176 (15.3)	150 (85.2)	26 (14.8)	
≥30 (pack-years)	165 (14.4)	139 (84.2)	26 (15.8)	
Smoking status				<0.001
Never smoker	806 (70.3)	750 (93.1)	56 (6.9)	
Former smoker	124 (10.8)	111 (89.5)	13 (10.5)	
Current smoker	217 (18.9)	178 (82.0)	39 (18.0)	
Cooking				0.602
Yes	842 (73.4)	765 (90.9)	77 (9.1)	
No	305 (26.6)	274 (89.8)	31 (10.2)	
Use coal or firewood				0.187
Yes	507 (44.2)	453 (89.3)	54 (10.7)	
No	640 (55.8)	586 (91.6)	54 (8.4)	
Occupational dust				0.187
Yes	384 (33.5)	354 (34.1)	30 (27.8)	
No	763 (66.5)	685 (65.9)	78 (72.2)	
Sleep latency	15 (10,30)	15 (10,30)	15 (5.25, 30)	0.386
Sleep efficiency	0.89 (0.75, 1.00)	0.89 (0.75, 1.00)	0.91 (0.78, 1.00)	0.134
PSQI scores	5 (3,7)	5 (3,8)	4 (3,6)	0.027
Sleep quality				0.008
Good	681 (59.4)	604 (88.7)	77 (11.3)	
Poor	466 (40.6)	435 (93.3)	31 (6.7)	

### 3.4. Disease comorbidities of participants with/without COPD

A total of 25.1% of participants were with at least one comorbidity. As compared with non-COPD participants, there were no significant differences in disease comorbidities and the number of comorbidities in COPD patients (Table 4).

**Table 4:** Disease comorbidities of participants with/without COPD.

	**Total**	**Non-COPD**	**Mild to moderate COPD**	***P*** **-value**
Asthma				0.606
Yes	45 (3.9)	40 (88.9)	5 (11.1)	
No	1,102 (96.1)	999 (90.7)	103 (9.3)	
Tuberculosis				0.286
Yes	43 (3.7)	37 (86.0)	6 (14.0)	
No	1,104 (96.3)	1,002 (90.8)	102 (9.2)	
Lung cancer				0.670
Yes	17 (1.5)	15 (88.2)	2 (11.8)	
No	1,130 (98.5)	1,024 (90.6)	106 (9.4)	
Diabetes				0.290
Yes	163 (14.2)	144 (88.3)	19 (11.7)	
No	984 (85.8)	895 (91.0)	89 (9.0)	
Stroke				0.598
Yes	66 (5.8)	61 (92.4)	5 (7.6)	
No	1,081 (94.2)	978 (90.5)	103 (9.5)	
Number of comorbidities				0.383
0	859 (74.9)	784 (91.3)	75 (8.7)	
1	251 (21.9)	221 (88.0)	30 (27.8)	
2	31 (2.7)	29 (93.5)	2 (6.5)	
≥3	6 (0.6)	5 (83.3)	1 (16.7)	

Asthma, tuberculosis, and lung cancer used Fisher's exact test.

### 3.5. Multivariable analysis on influencing factors of COPD

In the multivariable logistic model (Table 5), among all potential risk factors, only older age, male sex, and good sleep quality were significantly associated with COPD prevalence.

**Table 5:** Multivariable-adjusted odds ratios for COPD.

	**OR**	**95%CI**
**Age**		
40–59	1.00	
60–69	2.47	(1.13,5.41)
≥70	3.85	(1.80,8.25)
**Sex**		
Female	1.00	
Male	2.16	(1.43,3.26)
**Sleep quality**		
Good	1.00	
Poor	0.58	(0.37,0.90)

Adjusted variables: age, sex, sleep quality, smoking pack-years, occupational dust exposure, coal or firewood use, and education level.

### 3.6. Comparison of lung function

**Figure 2:**
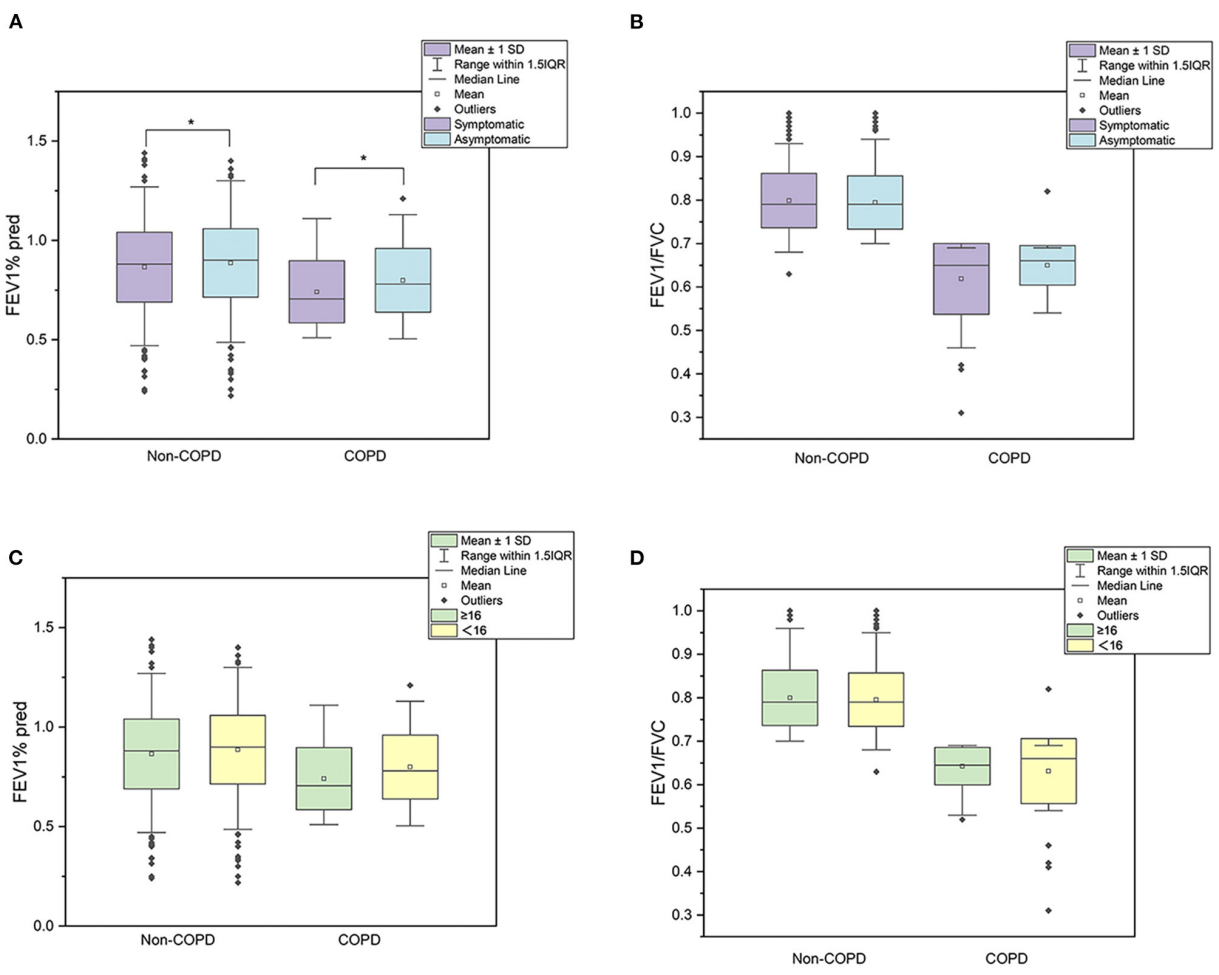
Lung function in different groups. **(A–D)** presented the mean 1 SD and 1.5IQR of lung function. **(A, B)** aboutFEV_1_%pred and FEV1/FVC grouped by symptoms, respectively. **(C, D)** about FEV_1_%pred and FEV1/FVC grouped by screening questionnaire scores.

Figure 2 shows lung function in different groups. Generally, participants with respiratory symptoms had lower FEV_1_%pred than asymptomatic participants in COPD and non-COPD groups. However, the value of FEV_1_/FVC was comparable between symptomatic and asymptomatic participants. According to the commonly used screening scale, FEV_1_/FVC and FEV_1_%pre did not significantly differ between participants who scored ≥16 or < 16 in either group.

The generalized linear model showed that FEV_1_%pred was correlated with older age (β = −0.062, *p* < 0.001), male sex (β = −0.031, *p* = 0.047) and respiratory symptoms (β = −0.025, *p* = 0.013) (Table 6). Sex, smoking, biomass exposure, BMI, and questionnaire score were not associated with FEV_1_%pred.

**Table 6:** A generalized linear model of FEV_1_%pred.

	**β**	***P*** **-value**	**95%CI**
**Age**			
40–59	0		
60–69	−0.025	0.076	(−0.052, −0.003)
≥70	−0.062	<0.001	(−0.090, 0.035)
**Sex**			
Female	0		
Male	−0.031	0.047	(−0.061, −0.000)
**Symptoms**			
No	0		
Yes	−0.025	0.013	(−0.045, 0.005)
**Smoking**			
Never smoke	0		
<30 (pack-years)	−0.019	0.349	(−0.059, 0.021)
≥30 (pack-years)	−0.021	0.256	(−0.056, 0.015)
**Occupational dust**			
No	0		
Yes	−0.016	0.125	(−0.005, 0.037)

## 4. Discussion

COPD is largely underestimated and underdiagnosed, where newly diagnosed patients mainly appear with mild to moderate disease (16). In the current study, only one-tenth of COPD patients had been previously diagnosed with COPD, and more than three-fourths of newly diagnosed COPD were mild to moderate patients. About half of these mild to moderate patients were asymptomatic, which might be related to the high missed diagnosis rate, since it is difficult to detect mild symptoms, especially when they do not interfere with daily life (17). Asymptomatic COPD is a universal occurrence among patients. For example, among 5,000 people included in the Third National Health and Nutrition Examination Survey in the US, 70% of those with undiagnosed COPD denied having cough or phlegm (18). According to COPD surveillance of Chinese residents in 2014–2015, 66.5% of 9,120 COPD patients had no respiratory symptoms (19).

Our multivariate analysis revealed no significant differences in symptoms and lifestyle factors between newly diagnosed COPD patients and non-COPD participants, which is consistent with some other studies. A study of 678 people in Sousse reported no significant differences in education, family history of COPD, chronic cough and phlegm, dyspnea, and diabetes between newly diagnosed COPD patients and non-COPD participants; however, COPD patients had lower BMI (20). Another study, which included 1,332 participants from the Canadian Cohort of Obstructive Lung Disease (CanCOLD) Study, reported no significant differences in mild-to-moderate cough and phlegm, sex, and numbers of comorbidities (21).

In the present study, older age, male sex, and sleep quality were significantly associated with newly diagnosed COPD, which are the emphasized questions in commonly used COPD screening scales. However, according to screening questionnaire scores, lung function did not significantly differ. Remarkably, symptomatic participants had worse lung function than asymptomatic participants, which indicated that symptoms other than sex and BMI had an important role in lung function. Similarly, a Swiss study, which included the general population, showed that individuals with cough, phlegm, or dyspnea had worse lung function (22).

The WHO definition of screening advocates that screening should be performed for common and treatable diseases with clear natural history. COPD is a widely recognized complex and heterogeneous disease (23). Spirometry is currently considered the gold standard for COPD screening due to its high sensitivity, specificity, and reproducibility (24). Yet, the fixed cut-off of FEV_1_/FVC commonly leads to over diagnosis of the elderly because of a faster decline in FEV_1_ than in FVC that occurs with age (25). In addition to an acceptable and effective screening method, an economic balance between the cost of identifying a case and acquired benefits is also essential. First, the price of a spirometer is high and cannot be afforded by most primary care clinicians (26). Moreover, it is further aggravated by the cost of post-bronchodilator spirometry and the training of physicians. Considering the huge population base in China, the cost of screening would be overwhelming. Besides, not all mild COPD patients progress to a more severe stage (27) and these people were at increased risks of anxiety and depression (28). A previous study revealed that some patients do not care to assess undiagnosed, especially asymptomatic diseases. GOLD notes no evidence that early spirometry screening of asymptomatic patients effectively improves COPD management or prognosis (7, 29, 30). Also, to date, the only known intervention to alter the natural history of COPD is smoking cessation. Only smoking cessation, oxygen therapy, and lung volume reduction surgery were beneficial in decreasing mortality (10, 31). Moreover, true-positive early diagnoses and false-positive reports can lead to irrational anxiety and depression (10). It is also important to consider potential treatment harms like pneumonia and decreased bone density with LABAs and corticosteroids (32).

The current situation concerning COPD screening is that physicians often pay low attention to respiratory symptoms, while some commonly used COPD screening questionnaires mainly focus on risk factors whilst ignoring symptoms (12, 14). Accordingly, the purpose of COPD screening should be seriously revised, including taking sex into account (33), it should be used to help undiagnosed patients with respiratory symptoms obtain early diagnosis rather than extensively screen the general population (34). Mild airflow restriction is not easily detected, especially by those individuals whose lifestyle does not include exercise. In addition, some patients attribute the symptoms as a normal consequence of old age or smoking, i.e., most patients who state being asymptomatic are actually symptomatic; however, they fail to recognize the symptoms (20). Therefore, physicians should pay more attention to respiratory symptoms and avoid the “don't ask, don't tell” strategies (34).

This is a large-scale study based on a community population. All participants were screened by trained physicians and post-bronchodilator spirometry, which strictly complied with the 2014 guidelines for pulmonary function examination. The study also has some limitations. Using fixed FEV_1_/FVC as a diagnostic criterion may result in over diagnosis of the elderly (35). Besides, the questionnaire was designed mainly for COPD screening. Therefore, only a few comorbidities were included and did not include hypertension which is a common chronic disease. And the comorbidities were self-reported. Therefore, it would underestimate the prevalence of comorbidities in COPD patients (36, 37). Additionally, the cross-sectional study cannot assess long-term outcomes due to its nature.

## 5. Conclusion

Newly diagnosed COPD patients in a community-based setting showed few differences from the general population, suggesting a targeted case finding strategy could be more beneficial than general screening. In addition, more attention should be paid to respiratory symptoms, developing more symptom-based screening tools, and exploring new therapies and interventions for COPD in the early stage.

## Data availability statement

The raw data supporting the conclusions of this article will be made available by the authors, without undue reservation.

## Ethics statement

The studies involving human participants were reviewed and approved by the Ethics Committee of Shanghai Fifth people's Hospital (2018 Ethics Approval No. 130). The patients/participants provided their written informed consent to participate in this study. Written informed consent was obtained from the individual(s) for the publication of any potentially identifiable images or data included in this article.

## Author contributions

Conceptualization: JS and NW. Formal analysis: XY and NW. Investigation: ZZ, YD, JL, SY, QX, and SH. Supervision: YZ, HD, JX, ZJ, and QJ. Writing—original draft: XY and ZZ. Writing—review and editing: XY, ZZ, JS, and NW. All authors have read and agreed to the published version of the manuscript.
